# GOLD stage-specific phenotyping of emphysema and airway disease using quantitative computed tomography

**DOI:** 10.3389/fmed.2023.1184784

**Published:** 2023-07-18

**Authors:** Philip Konietzke, Christian Brunner, Marilisa Konietzke, Willi Linus Wagner, Oliver Weinheimer, Claus Peter Heußel, Felix J. F. Herth, Franziska Trudzinski, Hans-Ulrich Kauczor, Mark Oliver Wielpütz

**Affiliations:** ^1^Department of Diagnostic and Interventional Radiology, University Hospital of Heidelberg, Heidelberg, Germany; ^2^Translational Lung Research Center Heidelberg (TLRC), German Center for Lung Research (DZL), University of Heidelberg, Heidelberg, Germany; ^3^Department of Diagnostic and Interventional Radiology with Nuclear Medicine, Thoraxklinik at University of Heidelberg, Heidelberg, Germany; ^4^Department of Pulmonology, Thoraxklinik at University of Heidelberg, Heidelberg, Germany

**Keywords:** chronic obstructive pulmonary disease (COPD), quantitative CT (QCT), GOLD stages, airway disease, lung emphysema

## Abstract

**Background:**

In chronic obstructive pulmonary disease (COPD) abnormal lung function is related to emphysema and airway obstruction, but their relative contribution in each GOLD-stage is not fully understood. In this study, we used quantitative computed tomography (QCT) parameters for phenotyping of emphysema and airway abnormalities, and to investigate the relative contribution of QCT emphysema and airway parameters to airflow limitation specifically in each GOLD stage.

**Methods:**

Non-contrast computed tomography (CT) of 492 patients with COPD former GOLD 0 COPD and COPD stages GOLD 1–4 were evaluated using fully automated software for quantitative CT. Total lung volume (TLV), emphysema index (EI), mean lung density (MLD), and airway wall thickness (WT), total diameter (TD), lumen area (LA), and wall percentage (WP) were calculated for the entire lung, as well as for all lung lobes separately. Results from the 3rd-8th airway generation were aggregated (WT_3-8_, TD_3-8_, LA_3-8_, WP_3-8_). All subjects underwent whole-body plethysmography (FEV1%pred, VC, RV, TLC).

**Results:**

EI was higher with increasing GOLD stages with 1.0 ± 1.8% in GOLD 0, 4.5 ± 9.9% in GOLD 1, 19.4 ± 15.8% in GOLD 2, 32.7 ± 13.4% in GOLD 3 and 41.4 ± 10.0% in GOLD 4 subjects (*p* < 0.001). WP_3-8_ showed no essential differences between GOLD 0 and GOLD 1, tended to be higher in GOLD 2 with 52.4 ± 7.2%, and was lower in GOLD 4 with 50.6 ± 5.9% (*p* = 0.010 – *p* = 0.960). In the upper lobes WP_3–8_ showed no significant differences between the GOLD stages (*p* = 0.824), while in the lower lobes the lowest WP_3-8_ was found in GOLD 0/1 with 49.9 ± 6.5%, while higher values were detected in GOLD 2 with 51.9 ± 6.4% and in GOLD 3/4 with 51.0 ± 6.0% (*p* < 0.05). In a multilinear regression analysis, the dependent variable FEV1%pred can be predicted by a combination of both the independent variables EI (*p* < 0.001) and WP_3–8_ (*p* < 0.001).

**Conclusion:**

QCT parameters showed a significant increase of emphysema from GOLD 0–4 COPD. Airway changes showed a different spatial pattern with higher values of relative wall thickness in the lower lobes until GOLD 2 and subsequent lower values in GOLD3/4, whereas there were no significant differences in the upper lobes. Both, EI and WP_5-8_ are independently correlated with lung function decline.

## Highlights

1. QCT showed significant differences between GOLD 0–4.
2. Airway parameters indicate a transient inflammatory response in GOLD 2 leading to airway destruction in GOLD 4.
3. QCT could detect spatial differences between upper and lower lung lobes.
4. FEV1%pred appears to be predicted by a linear combination of the independent variables EI and WP_3-8_.

## Introduction

Chronic obstructive pulmonary disease (COPD) is the fourth leading cause of death worldwide and typically results from prolonged inhalation of noxious particles (1, 2). The diagnosis is made by symptoms and pulmonary function testing (PFT), and severity is commonly classified according to the GOLD criteria (3). Early detection of the disease is of great interest because airway disease is potentially reversible with smoking cessation or appropriate treatment, thereby delaying irreversible disease progression (4, 5). PFT play a central role in COPD diagnosis, yet its role in early diagnosis and reproducibility are limited. FEV1 is often normal until more than 30% of lung tissue are damaged or more than 75% small airways are obstructed (6, 7) due to compensation mechanisms of the lungs. Koo et al. showed that airway changes such as thickened bronchiolar walls, decreased number of bronchioles, and narrowed bronchiolar lumen occur earlier than the presence of emphysema in patients with mild GOLD COPD (8).

The use of computed tomography (CT) has led to better characterization of disease heterogeneity across patients at risk of COPD (former GOLD 0) to late stages with advanced emphysema. CT imaging allows deeper insights into different disease phenotypes and more accurate assessment of disease severity and distribution (9–11). Moreover, software-based post-processing (QCT) may detect and quantify the presence and type of emphysema by analysis of lung density (12–14). The assessment of airway disease is more challenging and less validated (12). QCT of small airways disease (SAD) is particularly challenging because CT measurements of airways are accurate and reproducible up to an diameter of approximately 2 mm, but those airways may serve as a surrogate for the smaller airways (15, 16). All these parameters are heavily influenced by technical factors such as scanner type, contrast material, slice thickness, kernel, and post-processing software. However, larger airways that can be visualized on CT allow conclusions about the status of smaller airways (16, 17). At present, larger studies on the quantitative contribution of emphysema and airways disease to airflow limitation are sparse.

Thus, we hypothesized that emphysema and airway abnormalities are independent factors determining airflow limitation in COPD, and that both have different systematic impact in different GOLD stages. In this work-up, we used QCT for GOLD-stage specific differential characterization of emphysema and airway abnormalities in a relatively large group of 522 patients with former GOLD 0 COPD and GOLD stages COPD 1–4 were examined with a comparable protocol on the same scanner.

## Materials and methods

### Patients

This retrospective study was approved by the institutional ethics committee (S-646/2016). The patient cohort was retrospectively recruited from the institutional imaging database. All adult patients who had an inspiratory CT scan between 08/2016 and 01/2020 at our chest-hospital were included if whole-body plethysmography was available within 45 days. The clinical exclusion criteria were pulmonary infections, lung tumors >1 cm, prior lung surgery, or volume-reducing procedures, and technical exclusion criteria were use of contrast media, image artifacts, missing or other reconstructions as I40f\3 and I70f\3, slice thickness of 1.25 mm, or errors in data export. All examinations were visually inspected for the absence of significant motion artifacts and inclusion of all parts of the chest by a senior chest radiologist.

All patients were diagnosed with COPD according to GOLD 2020 (18). In addition to subjects of the GOLD 1–4 categories, smokers and former smokers with no assignable GOLD category including the “former GOLD 0” were enrolled. The “former GOLD 0” group includes subjects with normal PFT in terms of FEV1/FVC (ratio between forced expiratory volume in 1 s and forced vital capacity) but with COPD-specific symptoms. Based on these criteria, 5 study groups (GOLD 0–4) were defined.

### Whole-body plethysmography

All patients had whole-body plethysmography 0–45 days within the CT scan using reference values according to the Global Lung Initiative (19). In this study, vital capacity (VC), forced vital capacity (FVC), residual volume (RC), total lung capacity (TLC), forced expiratory volume in 1 s (FEV1), FEV1 predicted (FEV1%) and the ratio FEV1/FVC were used.

### CT acquisition and reconstruction

Non-contrast CT (Somatom Definition AS64, Siemens Healthineers AG) was performed in supine position, as recommended for COPD (20). All patients were instructed and carefully monitored for a stable full automatically instructed inspiratory breath-hold before scanning. The CT scanner was routinely calibrated every 3 months for water and daily for air. Scans were performed in caudocranial direction with a dose-modulated protocol (Caredose4D, Siemens Healthineers AG) using a reference of 120 kV and 70 mA or 100 kV and 117 mA (120/70//100/117 [kV/mA]; GOLD 0 = 28/95, GOLD 1 = 9/24, GOLD 2 = 44/71, GOLD 3 = 83/98, GOLD 4 = 16/24) at a collimation of 64 × 0.6 mm and a pitch of 1.45. The reconstructed slice thickness was 1.00 mm with 0.825 mm increment. For each patient, a set of two reconstructions was available, namely a medium soft reconstruction algorithm (I40f\3) and the edge-enhancing reconstruction algorithm (I70f\3).

### Quantitative post-processing

The in-house software YACTA, a non-commercial scientific software, segmented the airway tree and lung lobes fully automated, using the I40f\3 kernel for parenchymal analysis and the I70f\3 kernel for airway analysis as previously published (21–26). The total lung volume (TLV), emphysema index (EI) and mean lung density (MLD) were calculated for the total lung and for all lung lobes separately (right upper (RUL), middle (RML), and lower (RLL) lobe, as well as left upper lobe (LUL), lingula (LLi) and left lower lobe (LLL)). The airway parameters wall thickness (WT), total diameter (TD), lumen area (LA), and wall percentage (WP) were analysed generation-based in the trachea (G_1_), right and left main stem (G_2_), lobar (G_3_), segmental (G_4_), and the subsegmental bronchi (G_5-8_). Airway results were simplified by consolidating the generation-based results for the 3^rd^ to 8^th^ generation (G_3-8_).

### Statistical analysis

Statistical analyses were performed using R (R 3.3.2, Foundation for Statistical Computing) and SigmaPlot (Systat Software GmbH). Data are presented as mean ± standard deviation One-way analysis of variance (ANOVA), Tukey multiple pairwise-comparisons (Tukey Test), Spearman linear and multiple linear regression were used. Correlation coefficients were interpreted as follows: 0.00–0.10 (negligible), 0.10–0.39 (weak), 0.40–0.69 (moderate), 0.70–0.89 (strong), and 0.90–1.00 (very strong) (27). A value of *p* <0.05 was considered statistically significant.

## Results

### Patient cohort and demographics

The final cohort included 522 patients out of 15,631. 10,358 patients were excluded because of contrast media application, 4,503 patients because of clinical exclusion criteria. 275 patients were excluded due to technical exclusion criteria significant motion artifacts (74), incomplete inclusion of all parts of the chest (28), invalid export from PACS system (12), slice thickness 1.25 mm (27), missing I70f\3 reconstruction (77), other reconstructions as I40f\3 (conventional filtered backprojection) (21), B40f (11) and B40s (18) kernel. The final cohort consisted of 492 patients, of whom 29 patients were diagnosed with GOLD 0, 34 with GOLD 1 COPD, 123 with GOLD 2 COPD, 196 with GOLD 3 COPD, and 40 with GOLD 4 COPD.

**Figure 1:**
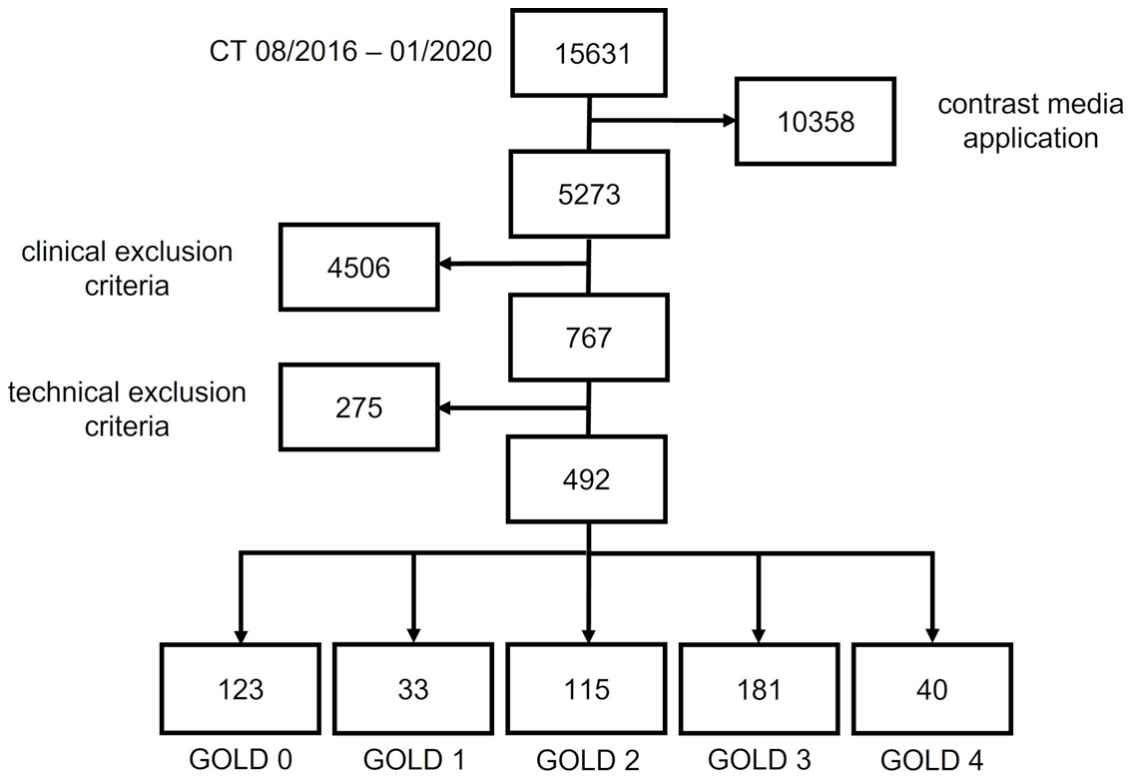
Patient recruitment flowchart.

Patients with GOLD 0 COPD had the highest vital capacity (VC) with 3.6 ± 1.2 L and the lowest residual volume (RV) with 2.6 ± 0.8 L as well as the lowest total lung capacity (TLC) with 6.2 ± 1.4 L. RV and TLC were higher in each GOLD stage form GOLD 0 to 3, being slightly lower again at GOLD 4 (all *p* < 0.001). FEV1%pred was lower at each GOLD stage as per definition (*p* < 0.001) (Figure 1; Table 1).

**Table 1:** Patient demograpics and body plerhysmography parameters.

	GOLD 0	GOLD 1	GOLD 2	GOLD 3	GOLD 4	pANOVA
*N*	123	33	115	181	40	-
Sex [f/m]	59/64	16/17	53/62	74/74	26/14	-
Age [y]	55 ± 15	63 ± 12*	63 ± 11	61 ± 10	64 ± 8	0.006
Height [cm]	1.71 ± 0.10	1.68 ± 0.11	1.68 ± 0.10	1.69 ± 0.08	1.66 ± 0.07	0.023
Weight [kg]	83 ± 19	79 ± 16	75 ± 18	72 ± 18	64 ± 12*	0.037
BMI	28 ± 5	28 ± 5	26 ± 5	25 ± 6	23 ± 4	0.001
TLC	6.2 ± 1.4	6.4 ± 1.7	7.8 ± 5.5	8.8 ± 4.5	8.1 ± 1.2	0.001
RV	2.6 ± 0.8	3.2 ± 1.5	4.8 ± 1.3*	5.8 ± 1.4*	5.4 ± 0.5	0.001
VC	3.6 ± 1.2	3.3 ± 1.2	2.4 ± 1.1*	2.7 ± 0.8	2.2 ± 0.8	0.001
FVC	3.5 ± 1.2	3.1 ± 1.2	2.2 ± 1.1*	2.3 ± 0.8	2.2 ± 0.9	0.001
FEV1 [L]	2.8 ± 0.9	2.2 ± 0.8	1.1 ± 0.6	0.9 ± 0.4	0.69 ± 0.23	<0.001
FEV1%pred	101.3 ± 6.4	88.3 ± 6.6	61.5 ± 9.7	41.7 ± 6.1	27.7 ± 2.3	<0.001
FEV1/FVC	0.8 ± 0.01	0.7 ± 0.1	0.5 ± 0.1	0.4 ± 0.1	0.28 ± 0.05	<0.001

Patient demographics (sex, age, height, weight, and BMI) and body plethysmography parameters total lung capacity (TLC), residual volume (RC), (vital capacity (VC) forced vital capacity (FVC), forced expiratory volume in 1 s (FEV1), FEV1 predicted (FEV1%), and FEV1/FVC ratio) are shown. All data are given as mean ± standard deviation. *p < 0.05 vs. previous GOLD stage.

### GOLD stage-specific quantification of emphysema and airway disease

**Figure 2:**
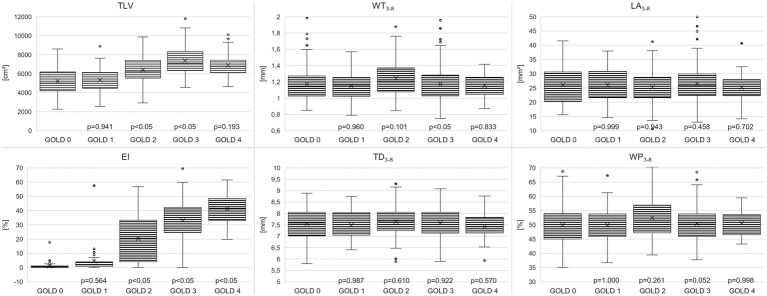
Boxplots for total lung volume (TLV), emphysema index (EI), wall thickness (WT_3-8_), total diameter (TD_3-8_), lumen area (LA_3-8_) and wall percentage (WP_3-8_) for the COPD stages 0–4. Box mark 25-75^th^ percentile, whiskers indicate 5^th^ and 95^th^ percentile, and individual outliers are given by black filled circles. Individual *p*-Values vs. previous GOLD stage are indicated.

**Figure 3:**
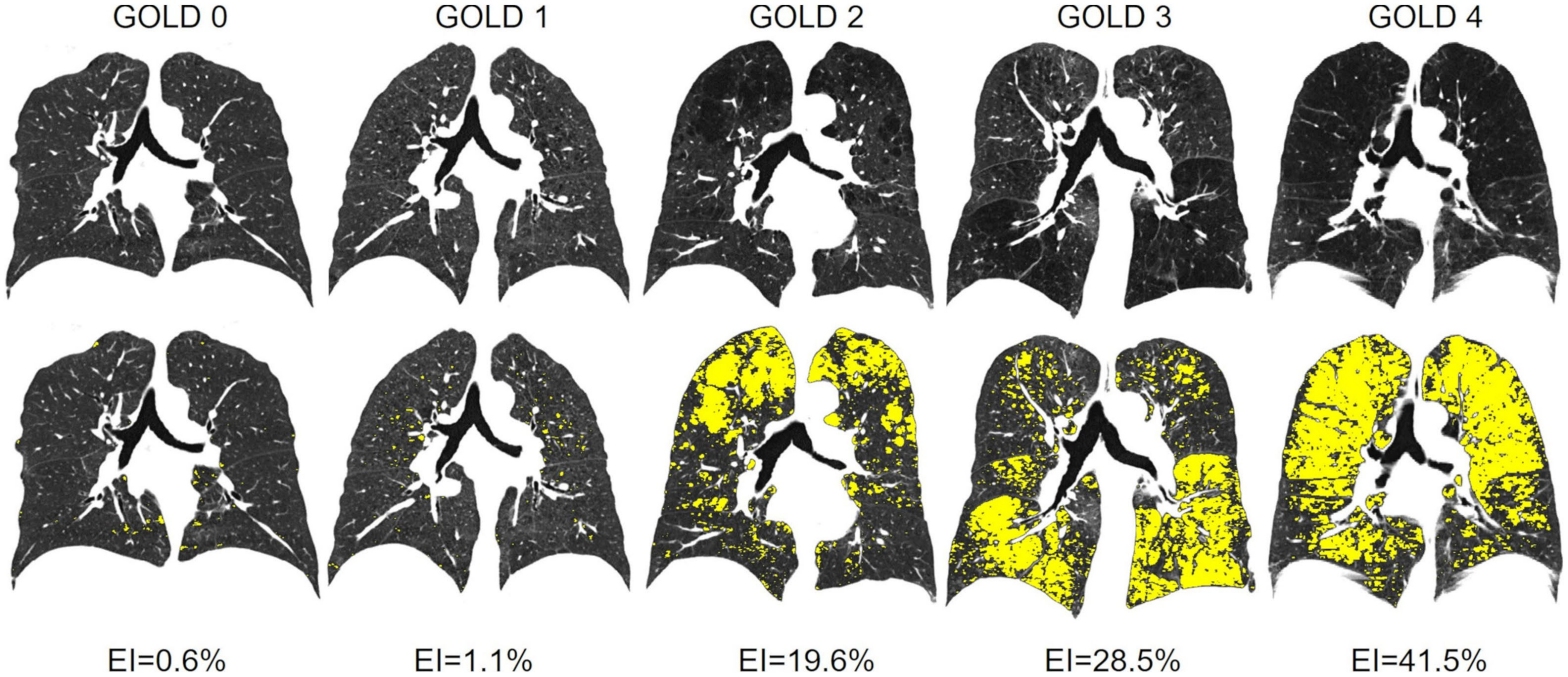
(Top line) Original CT images with increasing emphysema from GOLD 0 to GOLD 4. (Bottom line) Emphysema index (EI) parameter maps (emphysema = yellow) for the 5 different patients with GOLD 0 and GOLD 1 to 4 GOLD COPD.

TLV was 5198 ± 1349 cm^3^ in GOLD 0, higher with 7405 ± 1366 cm^3^ in GOLD 3 (*p* < 0.001) and lower in GOLD 4 with 6892 ± 1292 cm^3^ (*p* = 0.193). Emphysema was measured with an EI of 1.17 ± 1.8% in GOLD 0 and of 4.5 ± 9.9% in GOLD 1 (*p* = 0.564). EI was higher with increasing GOLD stages with 19.4 ± 15.8% in GOLD 2 (*p* < 0.05), 32.7 ± 13.4% in GOLD 3 (*p* < 0.05), and 41.4 ± 10.0% in GOLD 4 (*p* < 0.05). Accordingly, MLD showed a comparable pattern, with −802 ± 38 HU in GOLD 0 and progressively lower values in GOLD 1 with −820 ± 32 HU (*p* = 0.044), in GOLD 2 with −850 ± 30 HU (*p* < 0.05), and in GOLD 3 with −872 ± 19 HU (*p* < 0.05), while the MLD was again slightly higher in GOLD 4 with −876 ± 14 HU (*p* = 0.971) (Figures 2, 3; Table 2).

**Table 2:** QCT parameters for GOLD stages 0–4.

	GOLD 0	GOLD 1	GOLD 2	GOLD 3	GOLD 4	pANOVA
TLV [cm^3^]	5198 ± 1349	5401 ± 1286	6353 ± 1368	7405 ± 1366	6892 ± 1292	<0.001
EI [%]	1.0 ± 1.8	4.5 ± 9.9	19.4 ± 15.8	32.7 ± 13.4	41.4 ± 10.0*	<0.001
MLD [HU]	−802 ± 38	−820 ± 32*	−850 ± 30	−872 ± 19	−876 ± 14	<0.001
WT_3-8_ [mm]	1.17 ± 0.21	1.14 ± 0.16	1.24 ± 0.24	1.17 ± 0.19	1.15 ± 0.14	<0.05
TD_3-8_ [mm]	7.54 ± 0.68	7.47 ± 0.55	7.63 ± 0.64	7.30 ± 0.66	7.42 ± 0.54	0.328
LA_3-8_ [mm^2^]	26.05 ± 6.62	26.11 ± 6.07	25.47 ± 5.67	26.60 ± 6.05	25.16 ± 4.86	0.626
WP_3-8_ [%]	50.0 ± 7.2	49.8 ± 6.2	52.4 ± 7.2	50.3 ± 5.9	50.6 ± 4.6	<0.05

Total lung volume (TLV), emphysema index (EI) and mean lung density (MLD) as well as wall thickness (WT_3-8_), total diameter (TD_3-8_), lumen area (LA_3-8_) and wall percentage (WP_3-8_) were calculated. Generation-based results for WT, TD, LA, and WP were consolidated into one value (G_3-8_). All data are given as mean ± standard deviation. *p < 0.05 vs. previous GOLD stage.

**Figure 4:**
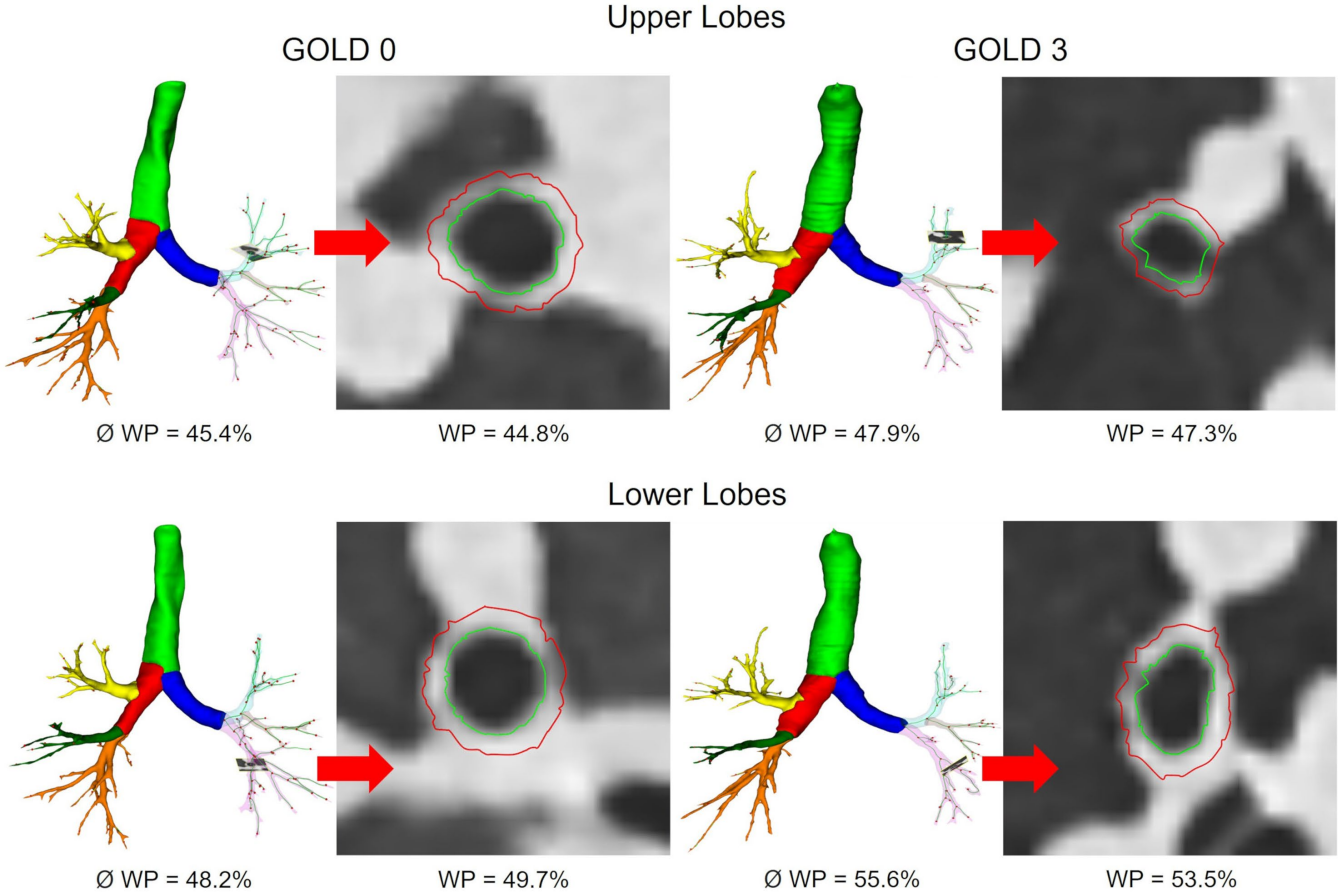
Segmented airway tree of the upper and lower lobes in GOLD 0 and GOLD 3. Average WP for the entire airway tree (Ø) and representative slices for individual bronchi are shown.

WT_3-8_ showed no essential differences between GOLD 0 and GOLD 1 with 1.17 ± 0.21 mm and 1.14 ± 0.16 mm (*p* = 0.960), respectively. In GOLD 2, it tended to be higher with 1.24 ± 0.24 mm (*p* = 0.101), while it was lower with 1.17 ± 0.19 mm in GOLD 3 (*p* < 0.05) and with 1.15 ± 0.14 mm in GOLD 4 (*p* = 0.833). WP_3-8_ showed a similar trend as there were no significant differences between GOLD 0 and GOLD 1, the highest values were found in GOLD 2, while the values in GOLD 3 and GOLD 4 were again lower. TD_3-8_ and LA_3-8_showed no significant differences between the GOLD stages (Figures 2, 4; Table 2).

### Lobe-based quantification of emphysema and airway disease

**Figure 5:**
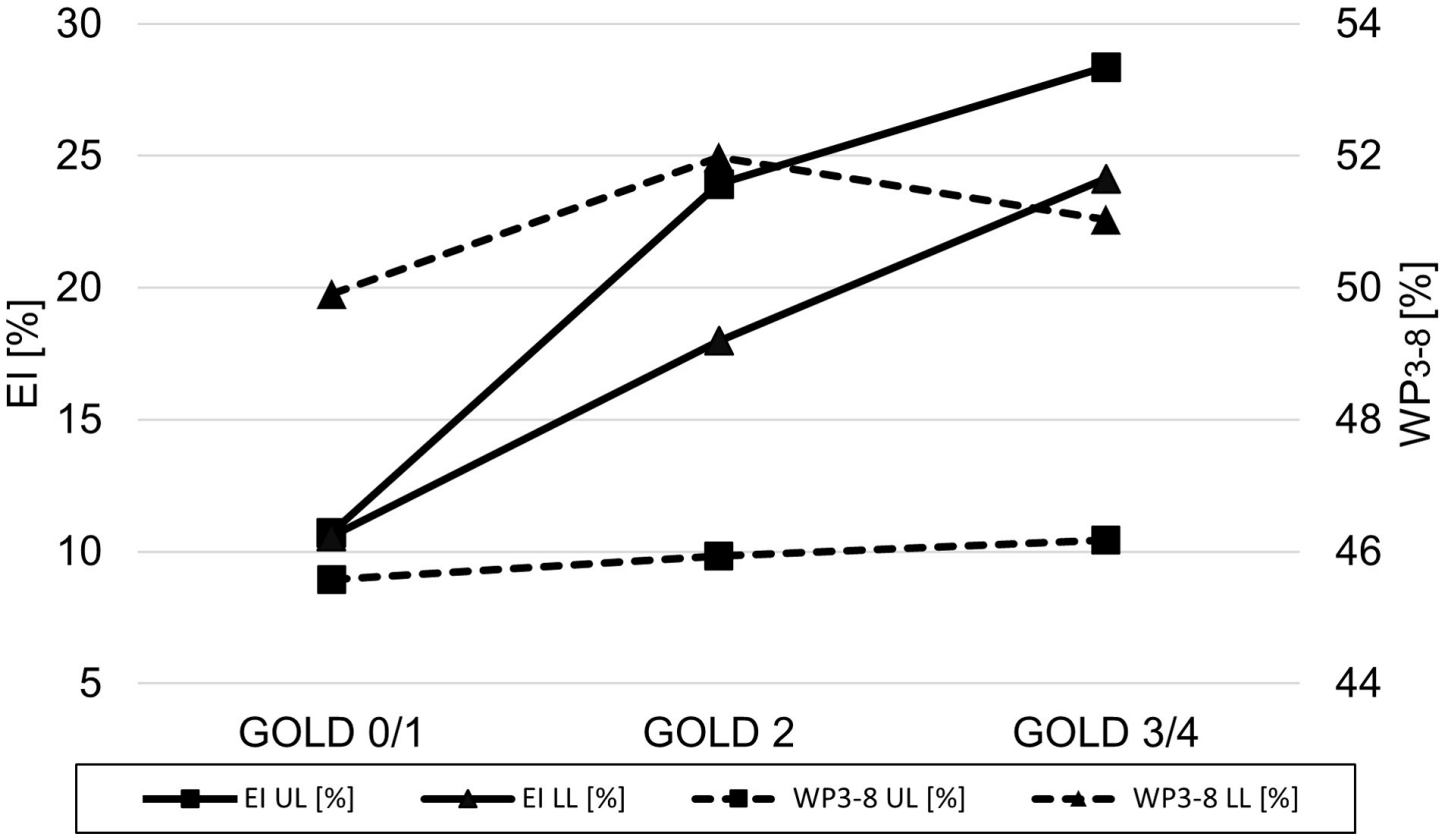
Line plot of emphysema index (▬ EI) and wall percentage (**− − -** WP_3-8_) for GOLD 0/1, GOLD 2, and GOLD 3/4 for the upper lobes (UL = square) and the lower lobes (LL = triangle).

A lobe-based analysis was performed, whereby only lobes with a complete set of results for the airway generations 3^rd^-8^th^ were included, which was the case for 913 out of the theoretical set of 2952 lobes. The lingula and middle lobe were excluded entirely due to a high number of missing results for individual generations. The lobe-based results for GOLD 0 and 1, and GOLD 3 and 4 were combined into GOLD0/1 and GOLD3/4, respectively, to compensate for limited complete datasets. The lobe volume difference between GOLD 0/1 and GOLD 3/4 was higher in the upper lobes with a difference of 288 cm^3^ (+20%), than in the lower lobes with a difference of 162 cm^3^ (+10%). The EI showed no significant differences between upper and lower lobes in GOLD 1, while EI was higher in the upper lobes in GOLD 2 and GOLD3/4. The values for WP_3-8_ showed different trends for the upper and lower lobes. In the upper lobes, WP_3-8_ was overall lower than in the lower lobes and tended to be higher with increasing GOLD stages (*p* = 0.887, *p* = 0.928). In the lower lobes, WP_3-8_ was 49.9 ± 6.5% in GOLD 0/1, higher in GOLD 2 with 51.9 ± 6.4% (*p* < 0.05) and tended to be lower in GOLD 3/4 with 51.0 ± 6.0%. In the upper lobes, TD_3-8_ and LA_3-8_ tended to be higher with higher GOLD stages (*p* = 0.201, *p* = 0.307). In the lower lobes LA_3-8_ showed no significant differences, while TD_3-8_ had the highest values at GOLD 2 (*p* = 0.216, *p* = 0.866) (Figure 5; Table 3).

**Table 3:** QCT parameters for the combined upper and lower lobes at different GOLD stages.

	GOLD 0/1	GOLD 2	GOLD 3/4	pANOVA
**Upper lobes**				
*N*	124	74	120	–
ULV [cm^3^]	1140 ± 387	1355 ± 437*	1428 ± 546	<0.001
EI [%]	11.0 ± 17.8	24.5 ± 17.6*	27.7 ± 22.5	<0.001
MLD [HU]	−833 ± 41	−864 ± 29*	−864 ± 39	<0.001
WT_3-8_ [mm]	1.10 ± 0.17	1.14 ± 0.21	1.15 ± 0.20	0.014
TD_3-8_ [mm]	7.86 ± 0.95	8.07 ± 1.07	8.25 ± 1.00	<0.05
LA_3-8_ [mm^2^]	32.01 ± 10.68	33.78 ± 10.58	36.40 ± 13.41	<0.05
WP_3-8_ [%]	45.6 ± 5.7	46.0 ± 5.0	46.0 ± 5.8	0.824
**Lower lobes**				
*N*	211	134	250	–
LLV [cm^3^]	1358 ± 422	1392 ± 476	1520 ± 482*	<0.001
EI [%]	11.0 ± 18.1	18.1 ± 17.2*	24.0 ± 19.1*	<0.001
MLD [HU]	−816 ± 51	−837 ± 41*	−845 ± 46	<0.001
WT_3-8_ [mm]	1.27 ± 0.20	1.35 ± 0.24	1.30 ± 0.23	<0.05
TD_3-8_ [mm]	8.33 ± 0.94	8.48 ± 0.95	8.41 ± 0.86	0.373
LA_3-8_ [mm^2^]	31.93 ± 9.98	31.79 ± 9.02	31.79 ± 8.14	0.924
WP_3-8_ [%]	49.9 ± 6.5	51.9 ± 6.4	51.0 ± 6.0	<0.05

Lobe volumes for the upper (ULV) and lower lobes (LLV), emphysema index (EI) and mean lung density (MLD), wall thickness (WT_3-8_), total diameter (TD_3-8_), lumen area (LA_3-8_), and wall percentage (WP_3-8_) are shown. All data are given as mean ± standard deviation. *p < 0.05 vs. previous GOLD stage.

### Emphysema and airway disease are independent factors of airflow limitation

TLC showed strong correlations with TLV for all GOLD stages (*r* = 0.79–0.86), low to moderate correlations with EI (*r* = 0.29–0.64), and weak to moderate correlations with WP_3-8_ (*r* = −0.10 - -0.51). RV showed strong to correlations with TLV and EI for GOLD 0–4 (*r* = 0.71, *r* = 0.77), while the correlations with WP_3-8_ were negligible to moderate (*r* = −0.04 - -0.54). The correlations between VC and TLV, EI and WP_3-8_ were overall weak for GOLD 0–4 (*r* = −0.13–0.18). The correlation of FEV1%pred for GOLD 0–4 showed moderate correlations with TLV (*r* = −0.59, *p* = 0.001) and strong correlations with EI (*r* = 0.78, *p* = 0.002), while the correlations for the individual GOLD stages were low to moderate (*r* = −0.10–0.47). The correlation between FEV1%pred and WP_3-8_ was negligible to moderate (*r* = 0.01–0.39) for GOLD 0–4 and all individual GOLD stages (Table 4; Supplementary Table 1).

**Table 4:** Spearman rank order correlation coefficient for lung function parameters and QCT.

	GOLD 0–4	GOLD 0	GOLD 1	GOLD 2	GOLD 3	GOLD 4
**TLC**						
TLV [cm^3^]	0.86 (0.001)	0.83 (0.001)	0.79 (0.001)	0.78 (0.001)	0.81 (0.001)	0.83 (0.001)
EI [%]	0.56 (0.001)	0.29 (0.001)	0.64 (0.001)	0.26 (0.001)	0.27 (0.001)	0.17 (0.399)
WP_3-8_ [%]	−0.12 (0.012)	−0.11 (0.245)	−0.51 (0.002)	−0.17 (0.078)	−0.10 (0.165)	−0.25 (0.211)
**RV**						
TLV [cm^3^]	0.71 (0.001)	0.38 (0.001)	0.48 (0.001)	0.56 (0.001)	0.65 (0.001)	0.43 (0.297)
EI [%]	0.77 (0.001)	0.10 (0.271)	0.69 (0.001)	0.56 (0.001)	0.35 (0.001)	0.49 (0.286)
WP_3-8_ [%]	−0.04 (0.386)	−0.07 (0.392)	−0.28 (0.118)	−0.16 (0.089)	−0.08 (0.252)	−0.54 (0.297)
**VC**						
TLV [cm^3^]	0.18 (0.001)	0.73 (0.001)	0.63 (0.001)	0.33 (0.001)	0.81 (0.001)	0.47 (0.001)
EI [%]	−0.38 (0.001)	0.25 (0.001)	0.1 (0.577)	−0.28 (0.001)	0.27 (0.001)	0.16 (0.331)
WP_3-8_ [%]	−0.13 (0.001)	−0.09 (0.329)	−0.29 (0.101)	−0.08 (0.411)	−0.11 (0.165)	−0.05 (0.725)
**FEV1%pred**						
TLV [cm^3^]	−0.59 (0.001)	−0.1 (0.271)	−0.02 (0.926)	−0.31 (0.001)	−0.15 (0.042)	0.26 (0.658)
EI [%]	−0.78 (0.001)	−0.16 (0.069)	−0.18 (0.311)	−0.43 (0.001)	−0.13 (0.076)	−0.71 (0.136)
WP_3-8_ [%]	0.01 (0.883)	0.01 (0.933)	0.39 (0.025)	0.04 (0.665)	0.02 (0.754)	0.31 (0.564)

Spearman rank order correlation coefficient were calculated for total lung volume (TLV), emphysema index (EI) and wall percentage (WP) with total lung capacity (TLC), residual volume (RV), vital capacity (VC) and FEV1%predicted (FEV1%pred).

**Figure 6:**
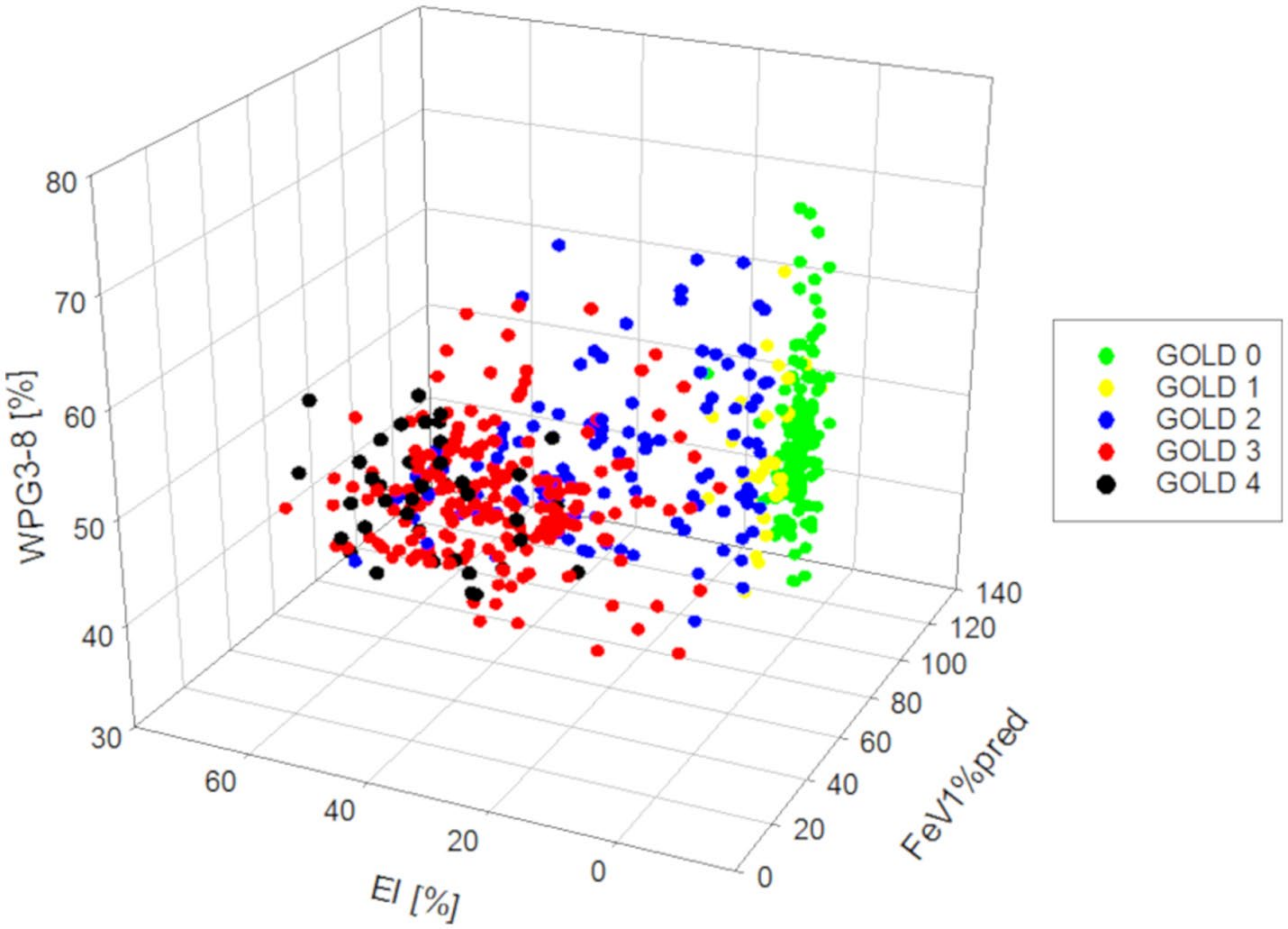
3D-residual scatterplots for the dependent variable FEV1%pred and the independent variables EI and WP_3-8._

Importantly, in a multilinear regression analysis the dependent variable FEV1%pred can be predicted by a combination of both the independent variables EI (*p* < 0.001) and WP_3-8_ (*p* < 0.001), whereas the independent variables biological sex (*p* = 0.893) and age (*p* = 0.598) could not (Figure 6; Table 4).

## Discussion

In this work, we demonstrate that emphysema and airway disease independently contribute to airflow limitation in COPD, in both high-risk patients and patients with COPD at GOLD stages 1–4, using QCT in 522 individuals studied with the same CT scanner and protocol. Second, we show that individual lobes show distinct differences of emphysema and airway disease at different GOLD stages with a relatively higher emphysema values in the upper lobes compared to the lower lobes. Thus, we found typical values of EI and WP for each GOLD-stages.

Total lung volume (TLV) was higher in GOLD 3 than in GOLD 0, which is within expectations as emphysema progression is associated with hyperinflation. However, TLV was again lower in GOLD 4, which may represent scarring in advanced destructive emphysema, but could also be related to increased mortality with a selection bias. The EI increased across all GOLD stages, but only significantly between GOLD 1 and GOLD 2, and GOLD 2 and GOLD 3, suggesting that the severity of the emphysema progression may not be linear. This contradicts the assumption that emphysema progression might be accelerated in advanced COPD, since locally severely altered alveolar micromechanics within an injured lung might itself ‘become an independent trigger of lung injury progression’ (29, 30). The explanation may be that in GOLD 4, emphysema is nevertheless higher in relation to the remaining normal lung tissue, although the absolute difference between GOLD 3 and GOLD 4 is lower. Overall, our results are consistent with the literature in which QCT has been successfully used to detect the progression of emphysema (31, 32). MLD increased also significantly from GOLD 0 to GOLD 4, as MLD and EI are strongly correlated. In this context, it should be mentioned that MLD in advanced COPD is influenced by both emphysema and lung fibrotic changes, the latter might explain the slightly higher MLD values in GOLD 4 when compared with GOLD 3. The use of the parameter Perc15 can be helpful to overcome this problem.

WT_3-8_ and WP_3-8_ showed no relevant differences between GOLD 0 and GOLD 1, most likely because the disease-related changes may be too subtle for detection. WT_3-8_ and WP_3-8_ showed the highest values at GOLD 2, accompanied by higher TD_3-8_ and a slightly lower LA_3-8_, which could be explained by inflammatory swelling of the airway walls in the process of bronchitis. This observation is supported by the literature, which recognizes small airway disease as a central feature of COPD, with increasing narrowing and destruction of the small airways representing a mixture of chronic inflammation (28, 33, 34). In this context, Koo et al. demonstrated significant loss of terminal and transitional bronchioles in patients with GOLD 1 and GOLD 2, as the remaining small airways had thickened walls and narrowed lumens that were also present in regions with no emphysema (8).

However, in contrast to parts of the literature, WT_3-8,_ WP_3-8_ and TD_3-8_ tended to be lower in GOLD 3 and GOLD 4, while LA_3-8_ was higher in GOLD 3 and again lower in GOLD 4. These changes may be interpreted as a possible transition from reversible airway inflammation to irreversible airway damage with airway wall degradation. This theory is supported by Smith et al., who observed significantly reduced airway wall thickness in most areas of the central tracheobronchial tree in COPD patients (35). They hypothesize that possible mechanisms include airway smooth muscle regression, apoptosis or replacement fibrosis resulting from chronic airway inflammation, and decreased bronchial vascular volume (36, 37). In addition, increasing emphysematous destruction of the lung parenchyma appears to have a significant impact on airway dimensions (38). In detail, emphysema leads to destruction of the lung parenchyma, resulting in loss of lung attachments that stabilize the airways and prevent them from collapse. Therefore, an increase in emphysema should result in partial airway collapse and thus a decrease in LA and TD, whereas WT and WP, unless the airway wall mass itself has not changed, should increase. On the other hand, hyperinflation leads to an increase in lung volume, stretching the airways and most likely increasing LA and TD and decreasing WT and WP, having a partially opposing effects on the airways. In our cohort, the reduction of LA_3-8_ in GOLD 4 might contribute to the destabilizing effects of emphysema.

We paid attention to potential regional differences within the lung by analysing individual lung lobes. EI was significantly higher in both upper lobes, resulting in greater volume increase due to emphysematous hyperinflation, consistent with the literature. (39). The spatial differences in airway involvement have been investigated in only a few studies. The COPDGene Investigators reported that there were no differences in airway wall thickness or Pi10 between patients with predominant lower and predominant upper lobe emphysema, although the former group had greater airflow limitation and more air-trapping (40). Park et al. showed that patients with predominant lower lobe emphysema showed greater airway involvement than those with predominant upper lobe emphysema, possibly leading to more frequent exacerbations and poorer response to therapy (41). In accordance with Park et al., our results also showed significant higher WP_3-8_ in the lower lobes. However, looking at the pooled results for GOLD 0/1, GOLD 2, and GOLD 3/4, we see that WP_3-8_ was higher in the upper lobes with increasing GOLD stages, while in the lower lobes it was the highest in GOLD 2 and again lower in GOLD 3/4. Interestingly, we would have expected a lower WP_3-8_ in higher GOLD stages in the upper lobes, as advanced emphysema should be associated with airway degeneration and collapse. One possible explanation may be the pooling of GOLD 3 and GOLD 4 data, which was necessary because airway segmentation becomes more difficult as disease progresses. Another reason could be the initial differences in WP_3-8_ between upper and lower lobes, which may influence the changes of airway dimensions in relation to the GOLD stage.

In our study cohort, strong correlations between parenchymal QCT and whole-body plethysmography parameters were observed. The QCT parameter TLV, which increased from GOLD stage 0 to 3, showed strong correlations with TLC determined by whole-body plethysmography, which has already been described (42). TLV showed also low to moderate correlations with EI as emphysema progression is associated with hyperinflation. The residual volume (RV) showed strong correlations with TLV and EI for GOLD 0–4, as the main volume that increases with COPD severity is RV (43). The correlations between vital capacity (VC) and the CT parameters TLV, EI were weak for GOLD 0–4. The correlation between FEV1%pred and TLV as well as EI was strong since its connection is well established in the literature (44). In comparison whole-body plethysmography parameters TLC, RC and VC showed at best weak correlations with QCT airway parameters. However, WP_3-8_ in GOLD 1 and EI in GOLD 4 appeared to correlate better with FEV1%pred, suggesting that FEV1 impairment may be more attributable to airway disease in the lower GOLD stages and more to emphysema in the higher GOLD stages. This may also be reflected in previous observations in the COPDGene study, demonstrating that small airways disease increases only from GOLD 0 to GOLD 4, and declines thereafter, whereas emphysema increases with every GOLD stage. Of note, results from larger airway analyses as in our present study can be regarded as surrogates for processes in the small airways not visible at CT (45). Restrictively, this observation may also be to selection bias since the patient numbers in GOLD 1 and GOLD 4 were relatively low. However, this observation also highlights the limitations of FEV1, as it is influenced by at least two very different pathophysiological mechanisms, namely airway wall thickening for inflammation (also in vessels) and attachment disruption (and vascular disruption) for protease activity in emphysema. In this study, this was also shown with multilinear regression, where the dependent variable FEV1%pred can be predicted by a linear combination of the two independent variables EI and WP_3-8_. There was no association with biological sex and age, which can be expected since FEV1% predicted is already normalized by the age, sex and other factors. However, emphysema, airway wall thickness (WT), total diameter (TD), and lumen area (LA) are dependent on sex and age (46, 47). In this context, it should be emphasized that other lung function parameters such as DCLO or FRC may be better suited to describe the different contributions of emphysema and airway disease, as already shown in the literature (48).

Our study has some limitations. The interpretation of quantitative parameters should be done carefully, as subtle changes may be due to noise or measurement errors. We tried to reduce technical confounders by using the same scanner, slice thickness, and reconstruction kernels. In our study cohort a dose-modulated protocol was used with a reference of 120 kV/70 mA (n = 180) or 100 kV/117 mA (n = 312), which may influence the results. However, two phantom based studies analysed the influence on the acquisition parameters current time product (mA) and tube potential (kV) on YACTAs QCT parameters (17, 49). The acquisition parameters used in the phantom study were not identical with 120 kV/60 mA and 80 kV/120 mA, but somehow comparable in their variance to each other. The comparison of the two protocols showed no significant differences, for example for MLD with −923.4 ± 6.4 HU vs. -923.2 ± 6.4 HU or for WP% with 43.3 ± 6.9% vs. 43.6 ± 7.2% (17, 49). However, the exact influence of the different protocols is difficult to quantify, and possible confounders cannot be completely excluded, although the use of both protocols in each GOLD group should weaken any possible effect. Other important confounders are the variation of lung volume and cigarette smoking status (9). The variation of lung volume was reduced as best as possible by instructing and monitoring all patients for a stable full inspiratory position. Smoking status was not considered because consistent smoking history was not available for all patients. This can be considered a substantial weakness, since cigarette smoking status influences the quantification of emphysema and airway disease (4, 50). Several authors have shown that current smokers have higher lung density, presumably due to a smoking-related increase in inflammatory cells in the lungs of current smokers, whereas other studies have shown that bronchial dimensions depend on current smoking status (51–54). However, in a large study from Rotterdam of approximately 2000 COPD patients, 41% were current smokers and 57.6% were former smokers or never smokers, indicating a relatively even distribution in an unselected population. Assuming a similar distribution in our study cohort, the impact of smoking on QCT parameters should be limited within each GOLD group. Although we agree that smoking status would have increased data quality, we still consider the results robust in the context of a descriptive cross-sectional study.

This work focused on QCT for GOLD stage-specific quantification of emphysema and airway disease. QCT parameters showed significant differences for GOLD 0–4 COPD, while lung lobe analysis revealed significant differences in the changes of airway dimensions between upper and lower lobes. The observed changes in airway dimensions may indicate airway inflammation in GOLD 2, which may lead to irreversible airway destruction in GOLD 3 and GOLD 4. Emphysema and airway disease both contribute independently to lung function decline. However, COPD is a very heterogeneous disease, which means that different stages of airway disease may coexist in the same lung, emphasizing the need to assess the spatial distribution within the lung.

## Data availability statement

The raw data supporting the conclusions of this article will be made available by the authors, without undue reservation.

## Ethics statement

The studies involving human participants were reviewed and approved by Ethics committee of the University of Heidelberg. The patients/participants provided their written informed consent to participate in this study.

## Author contributions

PK, CB, MK, WW, OW, CH, FH, FT, H-UK, and MW contributed study concept, design, and acquisition, analysis and interpretation of the data. OW developed the software used in this work. All authors contributed to the article and approved the submitted version.

## Funding

This study was supported by grants from the Bundesministerium für Bildung und Forschung (BMBF) to the German Center for Lung Research (DZL) (82DZL004A and 82DZL004A2). For the publication fee we acknowledge financial support by Deutsche Forschungsgemeinschaft within the funding programme Open Access Publikationskosten as well as by Heidelberg University.

## Conflict of interest

The authors declare that the research was conducted in the absence of any commercial or financial relationships that could be construed as a potential conflict of interest.

## Publisher’s note

All claims expressed in this article are solely those of the authors and do not necessarily represent those of their affiliated organizations, or those of the publisher, the editors and the reviewers. Any product that may be evaluated in this article, or claim that may be made by its manufacturer, is not guaranteed or endorsed by the publisher.

## Supplementary material

The Supplementary material for this article can be found online at: https://www.frontiersin.org/articles/10.3389/fmed.2023.1184784/full#supplementary-material

## Glossary

ANOVA: One-way analysis of variance
COPD: Chronic obstructive pulmonary disease
CT: omputed tomography
EI: mphysema index
FEV1: Forced Expiratory Pressure in 1 Second
FEV1%pre: FEV1 predicted
GOLD: Global Initiative for Obstructive Lung Disease
HU: ounsfield units
LA: Lumen area
LLi: Lingula
LLL: Left lower lobe
LUL: Left upper lobe
RML: Middle lobe
RLL: Right lower lobe
RUL: Right upper lobe
MLD: Mean lung density
PFT: Pulmonary function test
QCT: Quantitative computed tomography
RV: Residual volume
SAD: Small airway disease
TD: Total diameter
TLC: Total lung capacity
TLV: Total lung volume
VC: Vital capacity
WP: Wall percentage
WT: Wall thickness
