# Supplementary material for: GOLD stage-specific phenotyping of emphysema and airway disease using quantitative computed tomography
Source: Front Med (Lausanne). 2023 Jul 18;10:1184784. doi: 10.3389/fmed.2023.1184784 (PMC10393128; doi:10.3389/fmed.2023.1184784)
Supplement: Supplementary file 1 [file Table_1.docx]

Supplementary Table1. Spearman rank order correlation coefficient for lung function parameters and QCT

|  | GOLD 0-4 | GOLD 0 | GOLD 1 | GOLD 2 | GOLD 3 | GOLD 4 | GOLD 0-4 | GOLD 0 | GOLD 1 | GOLD 2 | GOLD 3 | GOLD 4 |
| --- | --- | --- | --- | --- | --- | --- | --- | --- | --- | --- | --- | --- |
| FEV1%pred | | | | | | | VC | | | | | |
| TLV [cm³] | -0.59 (0.001) | -0.1  (0.271) | -0.02  (0.926) | -0.31  (0.001) | -0.15  (0.042) | 0.26  (0.658) | 0.18  (0.001) | 0.73  (0.001) | 0.63  (0.001) | 0.33  (0.001) | 0.81  (0.001) | 0.47  (0.001) |
| EI [%] | -0.78  (0.001) | -0.16  (0.069) | -0.18  (0.311) | -0.43  (0.001) | -0.13  (0.076) | -0.71  (0.136) | -0.38  (0.001) | 0.25  (0.001) | 0.1  (0.577) | -0.28  (0.001) | 0.27  (0.001) | 0.16  (0.331) |
| MLD [HU] | 0.76  (0.001) | 0.10  (0.290) | 0.26  (0.135) | 0.43  (0.001) | 0.18  (0.016) | 0.31  (0.564) | 0.32  (0.001) | -0.25  (0.001) | -0.24  (0.175) | 0.24  (0.001) | -0.32  (0.001) | -0.23  (0.150) |
| WT_3-8_ [mm] | -0.05  (0.260) | 0.05  (0.573) | 0.53  (0.001) | 0.04  (0.655) | 0.05  (0.547) | 0.43  (0.419) | 0.01  (0.914) | 0.03  (0.803) | 0.15  (0.417) | 0.07  (0.403) | 0.07  (0.343) | 0.02  (0.907) |
| TD_3-8_ [mm] | -0.02  (0.691) | 0.04  (0.617) | 0.26  (0.144) | 0.02  (0.823) | 0.04  (0.564) | 0.08  (0.919) | 0.2  (0.001) | 0.27  (0.003) | 0.15  (0.395) | 0.29  (0.001) | 0.27  (0.001) | 0.27  (0.094) |
| LA_3-8_ [mm^2^] | -0.06  (0.237) | 0.04  (0.657) | 0.04  (0.843) | -0.04  (0.708) | -0.02  (0.783) | 0.14  (0.803) | 0.22  (0.001) | 0.29  (0.001) | 0.17  (0.348) | 0.30  (0.001) | 0.29  (0.001) | 0.33  (0.037) |
| WP_3-8_ [%] | 0.01  (0.883) | 0.01  (0.933) | 0.39  (0.025) | 0.04  (0.665) | 0.02  (0.754) | -0.26  (0.658) | -0.13  (0.001) | -0.09  (0.329) | -0.29  (0.101) | -0.08  (0.411) | -0.11  (0.165) | -0.05  (0.725) |
| RV | | | | | | | TLC | | | | | |
| TLV [cm³] | 0.71  (0.001) | 0.38  (0.001) | 0.48  (0.001) | 0.56  (0.001) | 0.65  (0.001) | 0.43  (0.297) | 0.86  (0.001) | 0.83  (0.001) | 0.79  (0.001) | 0.78  (0.001) | 0.81  (0.001) | 0.83  (0.001) |
| EI [%] | 0.77  (0.001) | 0.10  (0.271) | 0.73  (0.001) | 0.56  (0.001) | 0.35  (0.001) | 0.286  (0.49) | 0.56  (0.001) | 0.29  (0.001) | 0.64  (0.001) | 0.26  (0.001) | 0.27  (0.001) | 0.17  (0.399) |
| MLD [HU] | -0.76  (0.001) | -0.11  (0.235) | -0.48  (0.001) | -0.63  (0.001) | -0.39  (0.001) | -0.71  (0.054) | -0.58  (0.001) | -0.29  (0.001) | -0.55  (0.001) | -0.34  (0.001) | -0.32  (0.001) | -0.36  (0.062) |
| WT_3-8_ [mm] | -0.01  (0.852) | 0.01  (0.875) | -0.31  (0.084) | -0.12  (0.038) | 0.05  (0.508) | 0.03  (1.00) | 0.01  (0.860) | 0.05  (0.600) | -0.42  (0.001) | -0.04  (0.661) | 0.07  0.343 | -0.13  (0.520) |
| TD_3-8_ [mm] | 0.01  (0.001) | 0.16  (0.078) | 0.09  (0.594) | -0.15  (0.119) | 0.23  (0.001) | 0.26  (0.658) | 0.23  (0.001) | 0.33  (0.001) | 0.18  (0.320) | 0.12  (0.198) | 0.27  (0.001) | 0.22  (0.272) |
| LA_3-8_ [mm^2^] | 0.13  (0.001) | 0.19  (0.041) | 0.28  (0.108) | -0.02  (0.847) | 0.26  (0.001) | 0.43  (0.419) | 0.27  (0.001) | 0.35  (0.001) | 0.38  (0.029) | 0.13  (0.170) | 0.29  (0.001) | 0.24  (0.233) |
| WP_3-8_ [%] | -0.04  (0.386) | -0.07  (0.392) | -0.28  (0.118) | -0.16  (0.089) | -0.08  (0.252) | -0.54  (0.297) | -0.12  (0.012) | -0.11  (0.245) | -0.51  (0.002) | -0.17  (0.078) | -0.10  (0.165) | -0.25  (0.211) |

Total lung volume (TLV), emphysema index (EI), mean lung density (MLD), wall thickness (WT), total diameter (TD), lumen area (LA) and wall percentage (WP) are correlated with FEV1 predicted (FEV1%pred), (vital capacity (VC), residual volume (RC) and total lung capacity (TLC).
